# The consequences of reservoir host eradication on disease epidemiology in animal communities

**DOI:** 10.1038/emi.2016.46

**Published:** 2016-05-11

**Authors:** Farah Al-Shorbaji, Benjamin Roche, Rodolphe Gozlan, Robert Britton, Demetra Andreou

**Affiliations:** 1 Faculty of Science and Technology, Bournemouth University, Fern Barrow, Talbot Campus, Poole, Dorset, BH12 5BB, UK; 2 Unit for Mathematical and Computer Modelling of Complex Systems, Institute of Research for Development, 34394 Montpellier, France; 3 Pierre and Marie Curie University, 75005 Paris, France; 4 Biology of Aquatic Organisms and Ecosystems Research Unit, Institute of Research for Development, 34394 Montpellier, France; 5 National Museum of Natural History, 75231 Paris, France

**Keywords:** aquaculture, community ecology, emerging infectious diseases, epidemiological modelling, invasive species, topmouth gudgeon

## Abstract

Non-native species have often been linked with introduction of novel pathogens that spill over into native communities, and the amplification of the prevalence of native parasites. In the case of introduced generalist pathogens, their disease epidemiology in the extant communities remains poorly understood. Here, *Sphaerothecum destruens,* a generalist fungal-like fish pathogen with bi-modal transmission (direct and environmental) was used to characterise the biological drivers responsible for disease emergence in temperate fish communities. A range of biotic factors relating to both the pathogen and the surrounding host communities were used in a novel susceptible-exposed-infectious-recovered (SEIR) model to test how these factors affected disease epidemiology. These included: (i) pathogen prevalence in an introduced reservoir host (*Pseudorasbora parva*); (ii) the impact of reservoir host eradication and its timing and (iii) the density of potential hosts in surrounding communities and their connectedness. These were modelled across 23 combinations and indicated that the spill-over of pathogen propagules via environmental transmission resulted in rapid establishment in adjacent fish communities (<1 year). Although disease dynamics were initially driven by environmental transmission in these communities, once sufficient numbers of native hosts were infected, the disease dynamics were driven by intra-species transmission. Subsequent eradication of the introduced host, irrespective of its timing (after one, two or three years), had limited impact on the long-term disease dynamics among local fish communities. These outputs reinforced the importance of rapid detection and eradication of non-native species, in particular when such species are identified as healthy reservoirs of a generalist pathogen.

## INTRODUCTION

Introductions of non-native species into new regions are accelerating globally due to human-mediated activities including trade, food production and pest control.^1^ Non-native species can act as drivers of disease emergence^2, 3^ and can cause irreversible consequences for ecosystem functioning and services.^4, 5^ Although ‘enemy release' processes suggest that many pathogens will be lost during the introduction process, a small proportion do get released^6, 7^ and have the potential to spill over into novel hosts and lead to disease emergence.^8^ Recent examples of emergence of introduced diseases include White Nose Syndrome in bats,^9, 10^ snake fungal disease^11^ and the chytrid fungus in amphibians.^12, 13, 14^ While pathogen spill-over is closely associated with introduced species, it can also result from captive situations where species are farmed; aquaculture has been identified as a major source of new parasites and an amplifier of extant ones.^15, 16, 17^

After their introduction into animal communities, non-native generalist pathogens tend to have a higher probability of establishment than specialists as a result of a greater number of suitable hosts.^8, 18^ Furthermore, pathogens with environmentally transmitted propagules are also able to persist in the environment for prolonged periods prior to transmission, increasing their dispersal ability and infection of new populations.^13, 14^ This persistence has been observed in multiple high-profile pathogen systems, including cholera^19^ and avian influenza virus.^20^ Following introduction, new host populations provide additional opportunities for the development of persistent reservoirs of pathogen populations^21^ and thus can lead to subsequent episodes of disease emergence.^16, 22^ For example, animal reservoirs of human African trypanosomiasis (sleeping sickness) are expected to facilitate disease re-emergence in human populations even after the infection has been eradicated in the human population.^23^ Nevertheless, there are several community and physical factors that influence the ability of pathogens to spread and establish in neighbouring communities. These include the species composition, abundance, susceptibility and background mortality rate of new hosts, the interactions between these new host species and the initial pathogen prevalence (that is, the infection pressure).^24, 25, 26^ Species composition, host abundance and pathogen prevalence are well characterised factors in disease emergence, although not always considered in combination.^27, 28, 29^ It is expected that host background mortality could also influence the outcome of infectious outbreaks.^30, 31^ Host populations with high background mortality, due to their life history or a pre-existing infection, are predicted to be less able to recover following an epidemic.^26, 31^ How these factors interact to influence subsequent pathogen establishment and dynamics, and whether their effects are additive or synergistic, is poorly understood.

Management of pest species and diseases in spatially restricted systems, such as lakes, ponds and aquaculture systems, often involves chemical treatments to eradicate reservoir hosts in order to avoid future pathogen emergence.^32, 33^ In the case of host-specific pathogens, eradication can eliminate reservoir or intermediate hosts or vectors of the disease,^34^ as shown by malaria transmission rates being reduced via control of mosquito populations.^35^ Generalist parasites can be more difficult to eradicate given their potential presence in larger numbers of host populations, resulting in eradication having to target multiple species. Moreover, for generalist pathogens with environmentally transmitted propagules, there is a higher likelihood that the parasite also rapidly establishes in adjacent communities soon after its introduction.^20^ Recent experimental work on environmentally transmitted fish pathogens including *Flavobacterium columnare* supported this hypothesis, finding evidence of infections in communities downstream of infected hatcheries.^36^ The epidemiological complexity of generalist pathogens is difficult to characterise based solely on empirical data, and instead could be better understood using predictive epidemiological models that can be developed using existing data and be applied to predict outcomes of specific scenarios.^37, 38^

*Sphaerothecum destruens* is an intracellular, generalist fungal-like pathogen that has been identified as a potential threat to European fish biodiversity.^39, 40, 41^ *S. destruens* multiplies within host cells and releases spores into the environment through urine and seminal fluids,^40, 42^ which can zoosporulate in freshwater.^43^ Several cyprinid and salmonid fish species are susceptible to infection.^40^ Evidence suggests the pathogen was introduced into Europe via the accidental introduction of topmouth gudgeon *Pseudorasbora parva* from China in the 1950s, a healthy reservoir host that has since dispersed widely and achieved pan-continental distribution.^44, 45^ In the UK, *P. parva* invasion has been minimised via eradication of lentic populations to prevent their dispersal into lotic environments.^46^ Each eradication operation involved the application of the piscicide rotenone and to date have all been effective at extirpating *P. parva*. In this paper, *S. destruens* and *P. parva* (hereby referred to as the introduced reservoir host) were used as the model system.

The aim of this study was to examine potential factors affecting the establishment of a generalist pathogen following its environmental introduction from an adjacent source population, and to predict the consequences of its eradication from the source population on the adjacent community. Through modifying a recently developed epidemiological ecological model, the objectives were to investigate how disease establishment and its dynamics in the local communities were influenced by: (i) their physical proximity to the introduced reservoir host and the densities of susceptible native host species; (ii) the initial pathogen prevalence in the introduced reservoir host; (iii) inter-specific connectedness (that is, contact between species); (iv) the time-lag between the introduction of the reservoir host and its eradication; and (5) the role of species background mortality on the resilience of the local community.

## MATERIALS AND METHODS

The initial data used for developing the epidemiological single-host models^47^ were published experimental data from *S. destruens* infectivity trials.^39, 48, 49^ Epidemiological parameters were calibrated for several species including common carp *Cyprinus carpio* (low susceptibility), roach *Rutilus rutilus* (medium to high susceptibility), and sunbleak *Leucaspius delineatus* (high susceptibility). These species were selected for the model as they are the representative of the fish assemblages, for which *S. destruens* epidemiological data exists. The programme of *P. parva* eradication in the UK was used to model the removal of the introduced reservoir host population. For the purpose of this study it was assumed that all introduced hosts were eradicated and no infectious propagules reached local communities post-eradication.

### Multi-host susceptible-exposed-infected-recovered model

An epidemiological model was developed for *S. destruens* by extending the single-host susceptible-exposed-infectious-recovered (SEIR) model previously developed^47^ to a multi-species context^50, 51^ with the addition of demographic rates (birth *b* and natural mortality *m*) and inter-species contact rates. All the assumptions relevant to epidemiological processes at a species level, especially the dose-dependent transitions between categories, are discussed in Al-Shorbaji *et al.*^47^

In this model, susceptible individuals of species *i* could become infected by contacting infectious spores through direct contact (*β*) with infectious hosts from species *i* or *j* or indirectly through ingesting free-living spores (*ɛ_i_*(*Z*/*Z*+*k_ei_*)). Upon infection, an individual of species *i* became exposed (*E_i_*), meaning it has been infected but is not yet infectious. They moved to the Infectious class (*I*_*i*_), where they could release spores and transmit the pathogen to other hosts, through a rate corresponding to the inverse of the pathogen incubation period in species *i* (*σ_i_*(*Z*/*Z*+*k_si_*)). Infectious individuals remained infectious until they died (with rate *α_i_* *Z*/*Z*+*k_ai_*)) or recovered (*R_i_*) with rate *γ_i_*(*Z*/*Z*+*k_gi_*). In the experimental trials^40^ it was impossible to distinguish between hosts that were immune or recovered, so no distinction was made in the model as well. Therefore, it was assumed that recovered individuals could not be re-infected. Finally, the number of infectious propagules (*Z*) in the environment was the sum of infectious propagules released by each infectious individual per day (*Φ*). Zoospore concentration decreased with clearance rate *μ* (Equation 5). All the equations are described below:


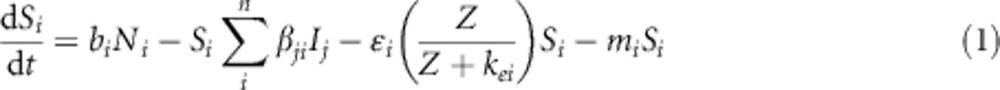



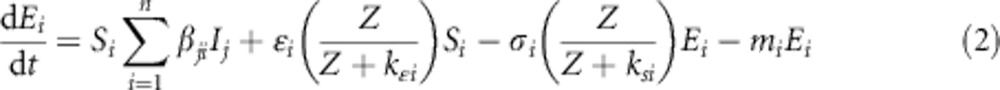



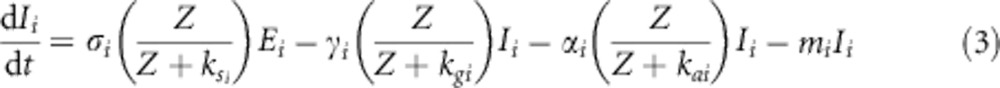



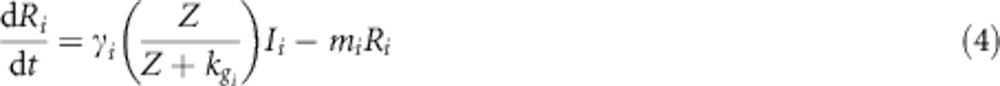



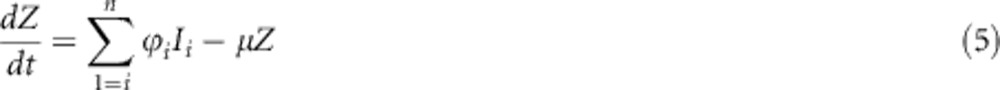


### Parameter estimation

For each species, epidemiological parameter values (direct (*β*) and environmental transmission (*ɛ*), incubation rate (*σ*), recovery rate (*γ*), infection threshold levels (*K*) and mortality from infection (*α*)) were based on previous single-host estimations.^47^ Environmental transmission (*ɛ*) was kept consistent across species, as the experimental values were within a similar range of uncertainty (*ɛ*=0.0007). For direct transmission *β* there were non-overlapping parameter ranges for each species, and the values were thus kept consistent with the minimum values of the ranges found empirically for each species. This minimised any estimation bias resulting from experimental systems with hosts in close proximity, while also remaining an accurate estimation based on the available data. Incubation, recovery and mortality from infection rates were also kept consistent with those found in the single host models. Conversely, the magnitude of interspecific direct transmission (*β_ji_*) between each species *i* and *j* was explored in this work while density dependent birth (*b*) and natural mortality rates (*m*) were estimated based on existing population data.^52^ All parameter ranges are shown in Table 1.

Spore durability (*μ*) was estimated based on experimental data on spores in sterile water.^43^ The estimation in Al-Shorbaji *et al.*^47^ represented a significantly shorter lifespan, due to experimental setups that used a continuous filtering system of the tanks (therefore the spores were rapidly flushed from the system). Here, the selected value was based on cold water temperatures (4°C–10 °C). *L. delineatus* was the only species with available data in both a single host setting and in cohabitation with the introduced reservoir host, *P. parva*^39^ (Supplementary Figure S1). Spore release (*φ*, the number of spores produced by one infectious individual per day) was estimated from the empirical data in both settings and compared. The estimated rate of spore release in the cohabitation model was 34% of the estimated value in the single host model (Supplementary Figure S1). This adjustment was thus applied to single host values of spore release for all species, as the cohabitation setting more closely represented natural conditions. Specifically, the cohabitation infections occurred through exposure to spores naturally released by *P. parva*, while the single host experiments flooded the fish tank systems with millions of spores. The resulting values were further reduced by two-thirds to simulate loss of spores due to water currents, attachment to sediments and predation.^53^

There was little sensitivity for threshold values (*K*) in the single-host models based on bath immersion experiments, resulting in ranges from 0 to 3 × 10^6^. However, the models for *L. delineatus* showed sensitivity to these parameters due to the incorporation of alternate infection methods in the model parameterisation. Therefore, the saturation thresholds for all susceptible species were kept consistent with *L. delineatus* values determined from the single host and cohabitation models. This was done for simplicity and consistency across species, and is shown in Table 1.

No competition between host species was included, but a population carrying capacity for each species was introduced to represent intra-specific competition, under the assumption that smaller species were more abundant than larger species^54^ (Supplementary Figure S2).

### Modelled scenarios

**Figure 1:**
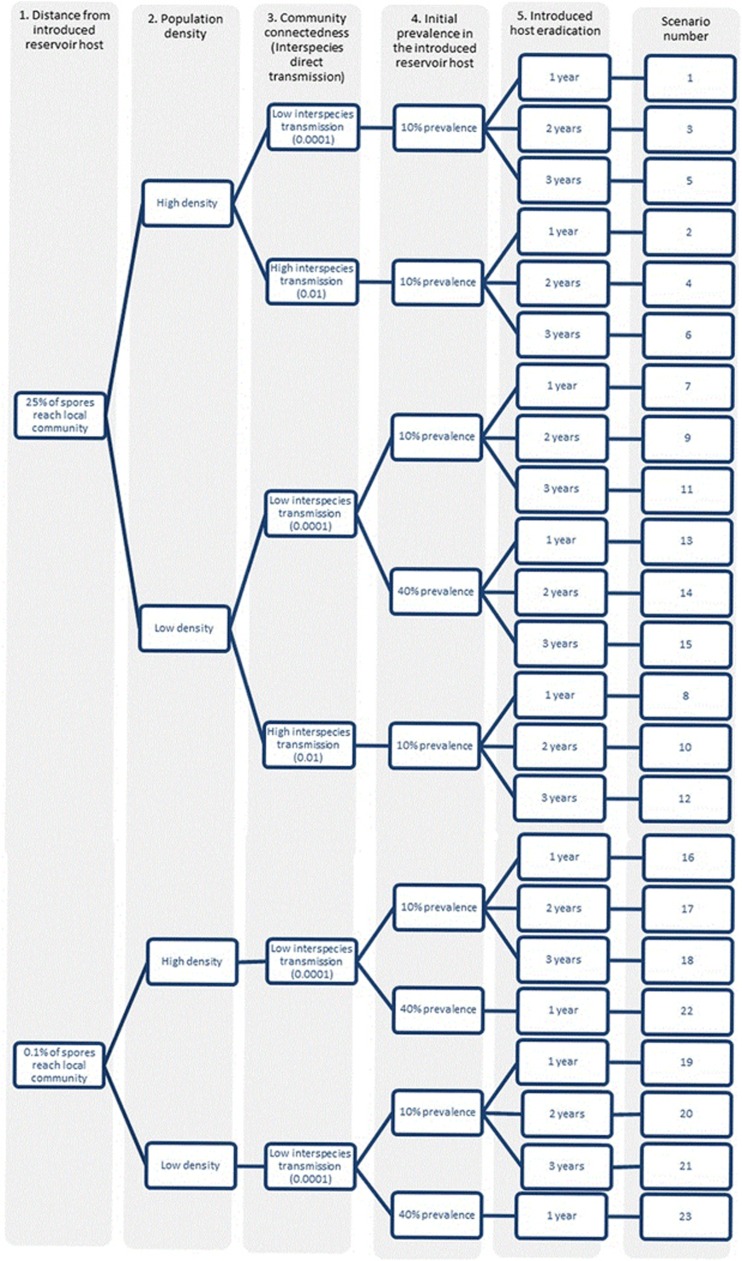
Each of the scenarios modelled in this work, testing various community factors on the establishment of a generalist pathogen via an introduced host. Columns 1–5 represent the main hypotheses tested, with the specific scenarios numbered. Furthermore, the role of high background mortality (**Hypothesis 6**) was tested by running all the scenarios again with a different natural mortality rate (10x the equilibrium mortality value).

Environmental pathogen transmission was modelled in a fish community, adjacent to an introduced *P. parva* population infected with *S. destruens*, which will be referred to as the local community and the source population, respectively. The introduced reservoir host population represented a source that could have been an aquaculture facility, a reservoir upstream of a dam or a fishing lake/pond. The local community included a species with low susceptibility to *S. destruens* (*C. carpio),* medium susceptibility (*R. rutilus*) and high susceptibility (*L. delineatus*). Each of the hypotheses listed below was tested using multiple combinations of parameter values (Figure 1) to investigate disease establishment and potential recovery of species following eradication of the reservoir host in the source population.

#### Hypothesis 1

The geographical distance between the reservoir host and local community was investigated for its influence on the force of infection (with more infectious propagules reaching communities that are closer to the reservoir host and vice versa). As such, communities in close proximity to the source population were subjected to 25% of *P. parva* produced spores (φ/4), and communities further away to only 0.1% of produced spores (φ/1000).

#### Hypothesis 2

The influence of disease prevalence in the introduced reservoir host was tested as a driver of disease emergence in the local community using 10 and 40% initial prevalence levels, which reflected published empirical data.^27^

#### Hypothesis 3

The impact of population density on disease dynamics was tested as a factor influencing disease dynamics. Low density populations were 50% of the values used as high densities; see Supplementary Figure S2 for details on population sizes.

#### Hypothesis 4

The influence of community connectedness (that is, how often species interacted with each other) was examined via interspecies direct transmission. In communities with a high degree of connectedness, interspecies direct transmission values were set at 0.01, marginally lower than intraspecific values. In communities where species did not interact with each other regularly, this interspecies transmission value was set at 0.0001, a value that indicated isolation between species.

#### Hypothesis 5

The reservoir host's source population was eradicated at one, two or three years post-introduction to test the effect of earlier vs later eradication. This was achieved by setting the introduced reservoir host population and the propagules they produced to 0 at a given time of eradication. This reflected the method used to eradicate *P. parva*, as it is assumed the rotenone would eliminate both host and pathogen from the habitat, and thus ensured that no infectious propagules would contact the local community post eradication.

#### Hypothesis 6

The local community's level of background mortality was tested for its effect on community response to introduced infection. All the scenarios (*n*=23) were repeated separately with a level of natural mortality ten times the equilibrium value estimated from observed data, for all species (Table 1).^52^ This represented communities that were experiencing significantly high levels of stress and thus could display a compromised immune response (that is, overcrowded fish farms that were already infected with a different pathogen^55, 56^). This allowed the testing of the impact of stress and multiple infections on the local community's response to introduced infection.

Multiple scenarios were run to test these hypotheses in all relevant combinations (detailed in Figure 1), to explore the additive effects (or not) of these factors. In each scenario, the local community was monitored for a total of 5000 days (~14 years), allowing the observation of long-term patterns. All simulations were run in R (Version 0.97.551, 2009–2014, Free Software Foundation (GNU Project), Boston, MA, USA). Shannon index was calculated for each community to observe changes in species diversity, using the *vegan* package (Version 2.3-3, Oulu, Finland)^57^ in R. The outputs of the simulations included the abundance of each species over time, including the number of individuals in each disease category (S, E, I and R) and the number of environmental propagules over time. Total abundance of each species over time was compared for significant differences between scenarios using Welch two sample *t*-tests and Kruskal–Wallis tests in the R stats package Version 2.15.3 (R Core Team, Boston, MA, USA).

## RESULTS

### Influence of geographical distance between the introduced reservoir and local community, and the effect of population density

**Figure 2:**
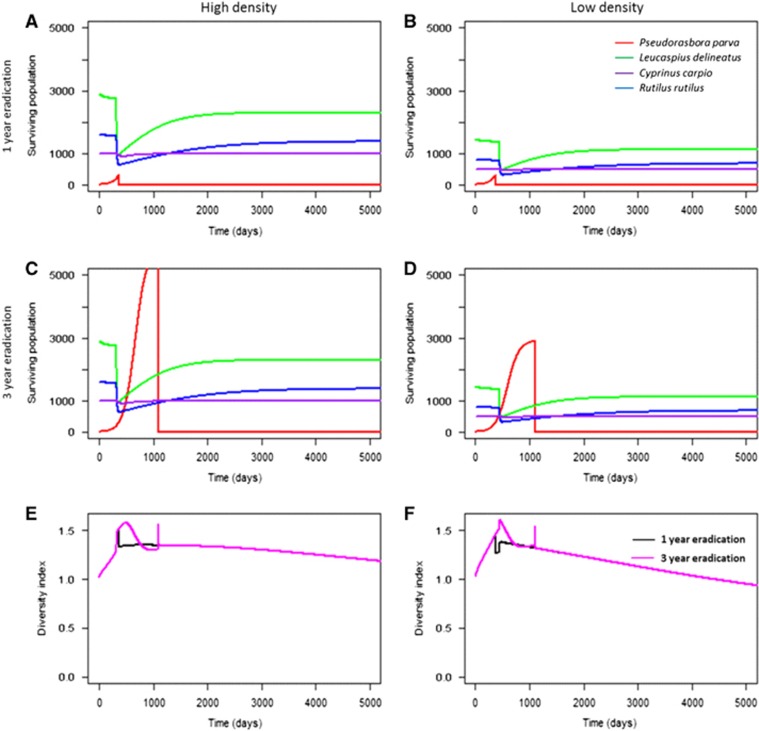
The abundance of each species in geographically distant communities at 1- and 3-year eradication of *Pseudorasbora parva* (**A−D**) and the corresponding Shannon diversity indices (**E−F**). Here, the role of population density (**Hypothesis 2**) and eradication time (**Hypothesis 5**) were tested. Higher population density accelerated the epidemic for *L. delineatus*, *R. rutilus* and *C. carpio* compared with lower density. The Shannon diversity index of each community at different times of eradication of *P. parva* demonstrated that high density communities maintained higher species diversity.

Greater geographical distance between the introduced reservoir host and the local community led to significantly delayed mortalities from infection in the local community (two sample *t*-test comparing the first 300 days after disease introduction: *t*=−16.94, degrees of freedom (d.f.)=1913, *P*<0.001), although total mortalities between the two scenarios were similar. In communities where 0.1% of the spores from the introduced reservoir host reached the local community at 10% prevalence (scenarios 16–21, see Figure 1), the epidemic was delayed by one to two years, as spores required a longer time to accumulate within susceptible hosts (Figure 2A and D). In all figures, eradication at two years was not shown for clarity. Overall, high density populations maintained higher levels of species diversity for a longer duration compared with low density populations (two sample *t*-test: *t*=78.9, d.f.=16 428, *P*<0.001), as evidenced by the community Shannon diversity index over time (Figures 2E and F; Supplementary Figure S2).

### Effect of the initial pathogen prevalence in the reservoir host population

**Figure 3:**
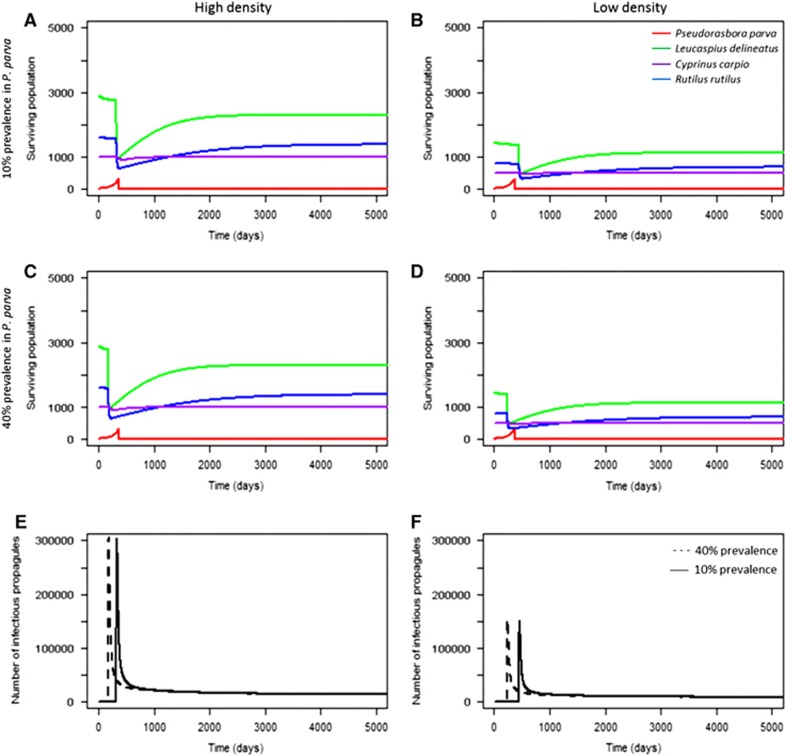
The abundance of each species (**A−D**) and corresponding *S. destruens* infectious propagule levels (**E−F**) over time at 10% (**A**, **D**) and 40% (**C**, **D**) initial prevalence of infection in *P. parva*. Here, the role of initial pathogen prevalence in the introduced host (**Hypothesis 4**) was tested. High initial prevalence accelerated the decline of *L. delineatus*, *R. rutilus* and *C. carpio* compared with 10% prevalence, although the local community declined to similar levels in both cases. The role of population density (**Hypothesis 2**) was also tested, demonstrating that high population density was correlated with higher levels of spores in the environment accumulating at a more rapid pace (**E**) compared with lower population densities (**F**).

**Figure 4:**
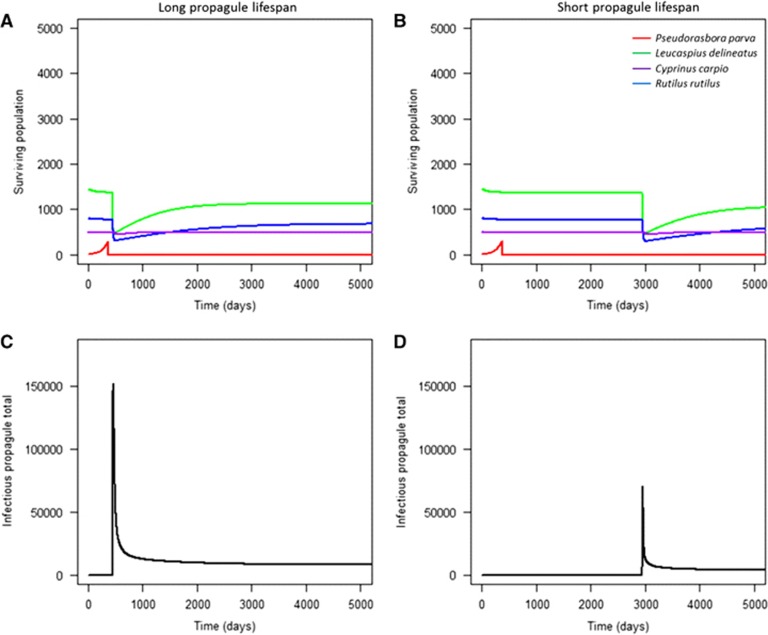
The effect of propagule lifespan on the local community's level of mortality. Longer propagule lifespan (14 days) accelerated mortality within the local community (**A**) compared with shorter propagule lifespan (five days) (**B**), and resulted in higher levels of propagules in the environment (**C**). Conversely, short propagule lifespan resulted in lower propagule levels in the environment (**D**).

For communities at a short geographical distance to the reservoir host population (scenarios 1−15), higher initial pathogen prevalence in the reservoir host did not significantly accelerate the rate of infection in the local community as high numbers of infectious propagules were already in contact with the susceptible species. However, pathogen prevalence in the reservoir host significantly influenced population decline in the community geographically further away from the source population (scenarios 16, 19, 22, 23; Figure 3). Higher initial pathogen prevalence in the reservoir host population led to accelerated mortalities from infection when compared with low prevalence (Figure 3A and D). The effect of initial pathogen prevalence was more pronounced in low-density populations, but was still significant in both high and low density local communities (two sample *t*-test (high density): *t*=2.94, d.f.=50 008, *P*<0.05; two sample *t*-test (low density): *t*=14.27, d.f.=49 912, *P*<0.001). This was expected, as higher initial prevalence would result in faster population growth of the pathogen, and thus a more rapid occurrence of secondary infections. This indicated that the abundance of infectious propagules in the environment drove community infection (that is, mortality) in susceptible hosts (Figures 3E and F). This was further tested by decreasing the infectious propagule lifespan to five days (the minimum duration of activity observed^43^). Decreasing propagule survival delayed mortalities in the local community by six to seven years, and reduced the total number of environmental propagules (Figure 4).

### Effect of reservoir host eradication time and geographical distance between communities

**Figure 5:**
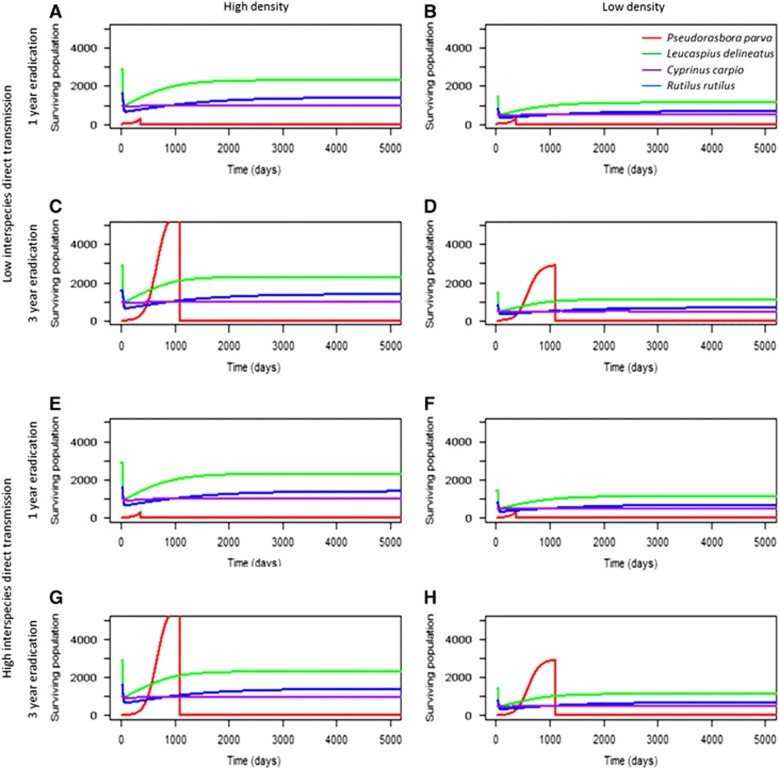
The abundance of each species at 1- and 3-year eradication of *P. parva* at high (**A**, **C**, **E** and **G**) and low population densities (**B**, **D**, **F** and **H**), and at low and high direct interspecies transmission (**A−D** and **E−H**, respectively). Here, the role of interspecies connectedness (**Hypothesis 3**) and eradication time (**Hypothesis 5**) were tested. For all tested scenarios, there was no significant effect of eradication time on *R. rutilus* abundance; however, there was a strong effect for *L. delineatus* and *C. carpio* abundance. Interspecies transmission had no significant effect on disease mortalities in local communities.

When the local community contacted 25% of infectious propagules from the introduced reservoir host (scenarios 1–12), the disease established in the local community within a year (Figure 5). All local communities recovered following the disease outbreak, irrespective of eradication time, although to lower levels compared with their starting population size. Following the initial environmental transmission from the introduced reservoir host to the local community, the disease dynamics in the local community were largely driven by intraspecific transmission. In *C. carpio* and *L. delineatus,* later eradication times (two and three years, respectively) led to higher mortalities in the 14 year period observed, especially in low density communities (Kruskal−Wallis test (*C. carpio*): *χ*^2^=53.61, *P*<0.001; (*L. delineatus*): *χ*^2^=9.47, *P*<0.05). However, over the timespan of 14 years *C. carpio* and *L. delineatus* achieved a similar equilibrium population size regardless of eradication time in each scenario.

**Figure 6:**
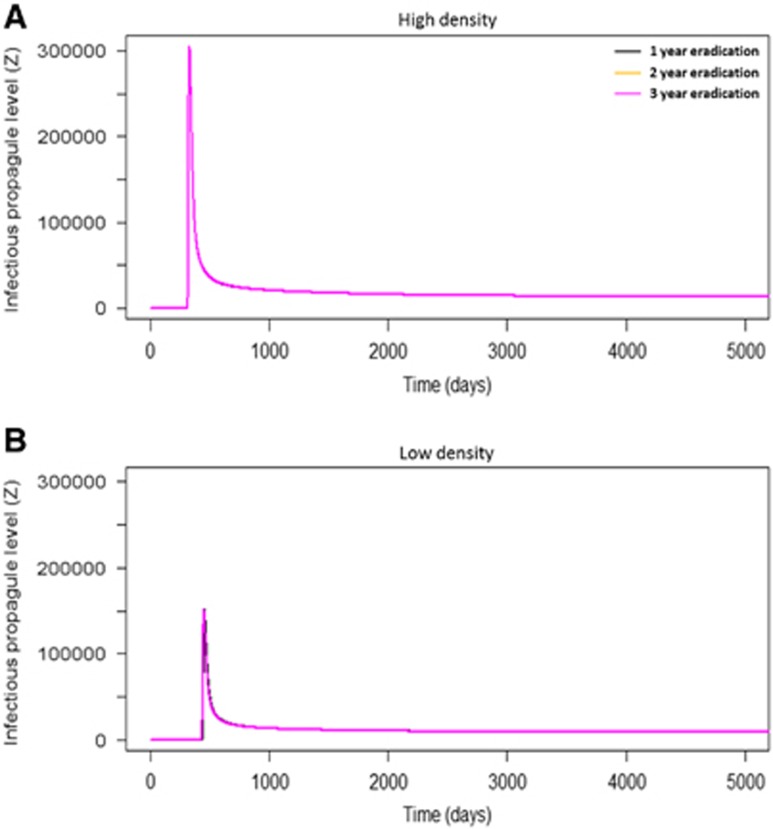
The number of infectious propagules in a community, at 1-, 2- and 3-year eradication of *P. parva* in high and low density communities. Here, the role of eradication time (**Hypothesis 5**) was examined more closely. In both high (**A**) and low (**B**) density communities, eradication time did not affect the number of infectious propagules in the system. Overall, low density populations had lower propagule numbers compared with high density populations.

**Figure 7:**
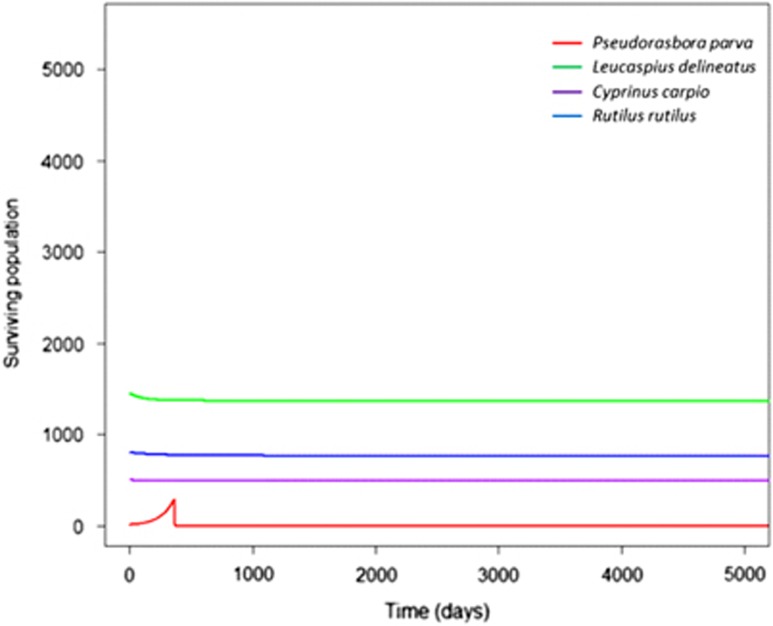
Excluding environmental transmission from *S. destruens*' transmission strategies prevented an epidemic from occurring in the local community, demonstrating the importance of environmental transmission in the pathogen's dispersal.

Eradication of *P. parva* at different times resulted in identical peaks of infectious propagule levels, although to higher levels in high density populations compared with low density (Figure 6). This suggested that the timing of eradication did not mitigate the local community's overall infection levels once the initial environmental introduction had occurred. Furthermore, in both high and low densities, after the outbreaks occurred the level of infectious propagules was maintained above 0, suggesting that the infection persisted in local communities at low levels. To demonstrate the key role of environmental transmission, the model excluded it (by setting *ɛ* to 0) (Figure 7), which prevented an epidemic from occurring in the local community.

### Effect of connectedness between species

Examining scenarios with 10% prevalence (1–12) in terms of their Shannon diversity index revealed that direct inter-specific transmission had no significant effect on local community mortality (two sample *t*-test: *t*=−0.04, d.f.=20 000, *P*>0.05).

### Effect of high background mortality on the resilience of the local community

**Figure 8:**
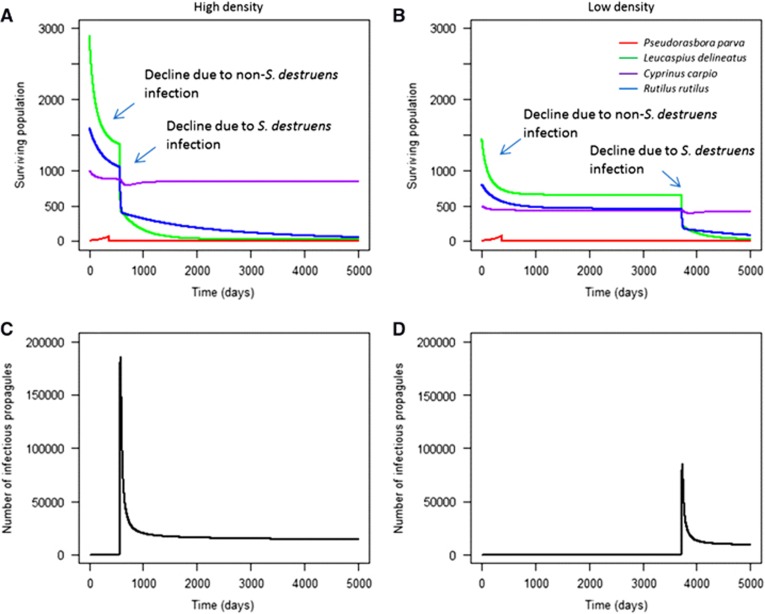
The effect of high background mortality on community recovery in high (**A**) and low (**B**) density communities, and the peaks in infectious propagule levels due to *S. destruens* outbreaks (**C**, **D**). The initial decline in species' abundance is a result of increased natural mortality (simulating an additional infection), followed by a decline from *S. destruens* infection at different times in high and low densities. Here, **Hypothesis 6** was tested, demonstrating that high background mortality can prevent susceptible species (*L. delineatus* and *R. rutilus*) in the local community from recovering after infection.

In scenarios with high background mortality due to previous infections, there was an initial population decline observed in the local community due to the magnified background mortality rate. Following this, each community experienced a further population decline due to infection with *S. destruens* (Figure 8). The timing of this second decline was dependent on the initial population density of the local community; high density populations led to significantly faster epidemics than low density populations (two sample *t*-test: *t*=−26.14, d.f.=9710, *P*<0.001).

In these communities, direct transmission between species affected the starting time of infection, although there was no significant effect on final species' abundance (two sample *t*-test: *t*=0.5, d.f.=50 007, *P*>0.05). Here, individuals could die before they began to release significant numbers of infectious propagules, so the relative contribution of direct transmission to pathogen expansion increased as the contribution of environmental transmission decreased. In high-density populations, the first infection following introduction was delayed by 11–17 days when direct interspecies transmission was low. This delay was 14–21 days in low-density populations. Following the outbreak, the level of infectious propagules remained above 0 in the local community, indicating that the infection could persist at low levels. Furthermore, susceptible species such as *R. rutilus* and *L. delineatus* remained at severely depleted population sizes, with no population growth observed. Less susceptible species such as *C. carpio* recovered to equilibrium population size.

## DISCUSSION

**Figure 9:**
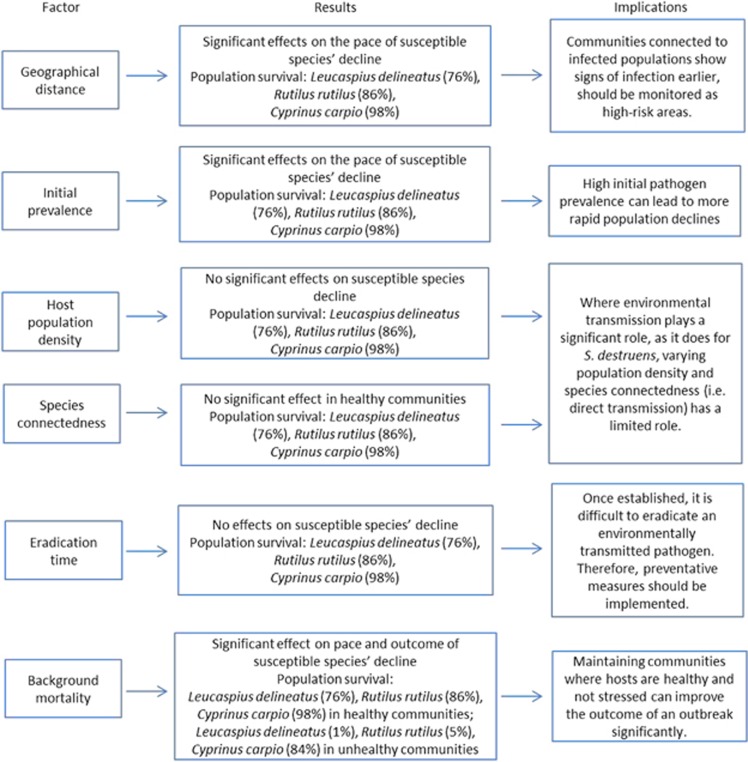
A summary of the important results of the SEIR simulation.

The release of environmentally transmitted propagules of a generalist pathogen such as *S. destruens* for less than a year was predicted to be sufficient for its establishment in local host communities. The disease dynamics in local communities were initially driven by environmental transmission of the parasite from the reservoir host population. The results demonstrated that without the presence of environmental transmission, the *S. destruens* epidemic would not have occurred in the local community. However, once there were multiple infected hosts in the local communities, the epidemic was no longer mediated by spill-over from the source population, but among susceptible host populations in the local community. In the long-term, both *L. delineatus* and *C. carpio* declined to similar levels in all eradication scenarios. These patterns were consistent in highly conservative scenarios where only a small proportion (0.1%) of the infectious propagules reached the local community. High background mortality in the local community (for example, due to multiple infections) prevented susceptible species' populations from recovering to sustainable levels. Overall, high density populations maintained higher levels of species diversity for a longer duration compared with low density populations. The results were summarised in Figure 9.

If *S. destruens* was present in natural systems, the predicted pattern of mortality suggests an initial rapid decline in the local communities after the introduction of the pathogen. The timing of this decline was dependent on the lifetime of the infectious propagules, which has important implications: as the lifetime of infectious propagules is dependent on temperature, changing climates could significantly impact the geographic range and prevalence of this infection in the environment. Following the initial decline, impacted populations would then recover and stabilise at a lower population density than their initial levels. In scenarios with high background mortality levels (that is, hosts with pre-existing infections), the results indicated that susceptible species could not recover. This has important implications for community resilience, suggesting that if infection was already present in the community and *S. destruens* was introduced, susceptible species could not recover to sustainable levels. Furthermore, surviving populations maintained the infection at a low prevalence, becoming reservoirs of infection themselves.^3^ The populations were sustained below the necessary density required for an infectious disease outbreak (that is, remained below the epidemic threshold^58^). However, if a pathogen can persist in multiple reservoir hosts, re-emergence of disease could occur if the populations cross the epidemic threshold, irrespective of eradication of the initial introduced host.

These risks are associated with pathogens with mixed transmission modes such as *S. destruens,* which operate under complex dynamics. Direct and environmental transmission act across two different time-frames; direct transmission indicates rapid infections driven by host contact rate, while environmental transmission takes place on a longer time scale with spores accumulating in the environment over time, leading to chronic pathogen exposure.^59^ In this system, environmental transmission played a key role in the dispersal of the pathogen between communities, facilitating the pathogen's initial emergence.^60^ Following this, direct transmission of virulent pathogens in the local community caused rapid declines in susceptible species' populations. After the mortalities occurred, infectious propagules remained in the environment at low levels, creating the potential for pathogen re-emergence.^61^ If the pathogen only used direct transmission, no spill-over would have occurred into the local community as pathogen dispersal would have been constrained in time. Conversely, if the pathogen only used environmental transmission, the outcome would have been chronic decline due to exposure to low environmental levels. Because *S. destruens* can use both methods of transmission, both high host mortalities and the potential for pathogen re-emergence from the environment are possible.

The continued persistence of a generalist pathogen in the environment and in local host communities could have substantial consequences for aquaculture and recreational fisheries.^62, 63^ As demonstrated here, eradication of the reservoir host is unlikely to eliminate the pathogen if it has already dispersed. The lag time between detection and eradication of an introduced species can be considerable due to issues of resources, legislative and policy requirements, and political will.^46^ For example, *P. parva* was first detected in UK aquaculture in the mid-1980s and in the wild in the mid-1990s, but the first eradication of a population (in a recreational fishery) was in 2005.^64^ Following eradication of free-living hosts, a common procedure is then to re-stock with new fish in order to restore high population densities in both recreational and commercial settings.^65^ However, this work suggests that this would not prevent the re-emergence of the pathogen or even its continued spread into new systems, as the pathogen was maintained at low levels in reservoirs after the outbreak. In the shrimp farming industry, pathogen re-emergence from disease reservoirs has occurred multiple times after populations were restocked with new individuals.^63^ This has severe public health and financial consequences, particularly as the conditions in which many aquaculture facilities operate (that is, extremely high densities) can lead not only to emergence, but to an increase in pathogen virulence.^17, 31^ Thus, in disease management terms the benefit of eradicating the free-living species and/or subsequent restocking might be limited.

To date, *P. parva* has been introduced to numerous sites in Europe and North Africa, with several links to major river catchments.^45^ If these introduced populations carry *S. destruens* even at a low prevalence, they pose a potentially serious threat to native fish communities in surrounding water bodies. Here, it was predicted that environmental transmission was sufficient for pathogen establishment in neighbouring communities, and could lead to multiple reservoirs of infection. The bi-modal transmission of the pathogen increased the risk of pathogen emergence and persistence, which has profound long-term implications for the ecology and management of affected populations. The results and recommendations from this work can also be applied to other generalist pathogen systems with bi-modal transmission, such as the amphibian chytrid fungus *Batrachochytrium dendrobatidis*, and provide a new level of consideration in eradication protocols. For these pathogens, rapid diagnosis and if possible eradication is important to minimise mortalities, as this study demonstrated that even one year following introduction is sufficient for pathogen establishment. For terrestrial systems, it is expected that eradication would have a more significant impact on disease emergence, because hosts are not embedded in their environment as they are in aquatic systems, and the pathogen may not be established in an environmental reservoir. Developing tools such as eDNA detection or filtering procedures^66^ following animal transport can potentially prevent the initial emergence of a pathogen like *S. destruens*. In freshwater habitats, pathogens can also spread downstream and lead to native species decline, as predicted in this work and demonstrated empirically,^36^ which can have substantial economic and ecological consequences.

This work stressed the importance of using preventative measures against pathogen expansion and establishment, particularly for chronic generalist diseases, as reactive measures such as eradication may not work. Relevant measures can include emerging multi-host pathogens on notifiable parasite lists, especially if effective surveillance systems and response protocols are developed that facilitate early detection and the initiation of rapid actions to prevent their dispersal to adjacent communities. There are currently eight notifiable fish diseases in the UK, including *Gyrodactylus salaris*, a macro-parasite which causes highly virulent infections in Atlantic salmon *Salmo salar.*^67, 68^ Studies on the spread of this infection between populations in countries like Norway have provided a strong basis for the successful development and application of management strategies in the UK, such as heightened surveillance, development of early warning systems and, should an outbreak occur, monitoring of dispersal in the wild.^69, 70^ Furthermore, this work has demonstrated that maintaining healthy populations in general is critical for population recovery. This example demonstrates the utility of using existing knowledge of a pathogen to design and implement robust management programmes to minimise the chance of disease outbreaks, and the actions needed to limit their potential spread. Such approaches could have a strong application to the management of more novel and generalist pathogens such as *S. destruens*.

## Figures and Tables

**Table 1:** Parameter values for all species, for communities in close proximity (CP) and relatively far proximity (FP) from the introduced reservoir host

**Parameter (per day)**	**Leucaspius delineatus**	**Cyprinus carpio**	**Rutilus rutilus**	**Pseudorasbora parva**
*β* (direct transmission)	0.015–0.020	0.016–0.017	0.080–0.100	0.011
*α* (mortality from infection)	0.220–0.300	0.017–0.025	0.129–0.130	0
*ɛ* (environmental transmission)	0.0007	0.0007	0.0007	0.0007
*σ* (incubation rate)	0.230–0.233	0.013–0.081	0.095–0.11	0.072
*γ* (recovery rate)	0.140–0.170	0.065–0.072	0.099–0.101	0.108
*K_e_* (threshold for infection)	30000	30000	30000	30000
*K_a_* (threshold for mortality)	3000	3000	3000	3000
*K_g_*(threshold for recovery)	6500	6500	6500	6500
*K_s_* (threshold for incubation)	7200	7200	7200	7200
*μ* (spore mortality)	0.071	0.071	0.071	0.071
*φ* (spore release)	40	40	55	13 (CP); 0.050 (FP)
*b* (birth)	0.010	0.011	0.004	0.010
*m* (natural mortality; low level)	0.000430	0.000140	0.000170	0.000430
Population carrying capacity	1500 (low); 3000 (high)	500 (low); 1000 (high)	800 (low); 1600 (high)	3000 (low); 6000 (high)
