# Supplementary material for: The consequences of reservoir host eradication on disease epidemiology in animal communities
Source: Emerg Microbes Infect. 2016 May 11;5(5):e46–. doi: 10.1038/emi.2016.46 (PMC4893545; doi:10.1038/emi.2016.46)
Supplement: Supplementary Information 1 [file emi201646x1.pdf]

Parameter estimation continued

Experimental work has demonstrated several fish species are sensitive to *S. destruens* infection at varying levels of virulence, including roach *Rutilus rutilus*, common carp *Cyprinus carpio*, and sunbleak *Leucaspius delineatus*[1,2]. For all single host model outputs[3], there was a range of possible values for each parameter that did not significantly alter the model output's mean squared error (MSE) against the experimental data, suggesting that values within this rangewere equally plausible. The nature of the experimental setups where hosts are exposed to millions of spores restricted some parameters while leaving others with a wider range of possible values (i.e. the model's uncertainty). This is an unavoidable limitation of using empirical data, as experiments that overcome this difficulty are not possible. Sensitivity analyses showed that this parameter selection did not significantly affect model output.

Parameter values from the single host *L. delineatus* model were input into a two host model including introduced reservoir host *Pseudorasbora parva* and *L. delineatus*, which was then calibrated with experimental cohabitation data of these two species [1]. Even without direct contact between species (they were kept in separate tanks), the transfer of infectious propagules through the water column caused a severe *S. destruens* outbreak in *L. delineatus* (Figure S1). The rate of spore release for *L. delineatus* found in the single host model (350 per day) resulted in significantly faster mortalities in the two host system than those observed, so this parameter was optimised in addition to the *P. parva* parameters, testing for a value between 10 and 500. The value that resulted in the lowest MSE for the two host model output was 120 spores per day, 34% of the value in the single host model. This was expected as the experimental cohabitation did not artificially flood the model system with spores, but only included spores naturally released from *P. parva*. This adjustment was thus applied to the remaining species spore release values to simulate

naturally caused infections. The model was run for 100,000 iterations incorporating ± 25% random variation in each parameter in each iteration to test for parameter sensitivity and best fit values.

When all *P. parva* were assumed infected (100% disease prevalence), mortalities were marginally higher than the experimental data (Welch two sample *t*-test: $t = 1.74$, d.f. = 464, $P = 0.083$). This initial pathogen prevalence was systematically reduced by 10% until a best fit was achieved at 10% prevalence. Therefore, 10% was selected as the initial prevalence value, a conservative estimate compared with published values of 67%-74%[4]. However, given that the pathogen was not detected in *P. parva* used in the cohabitation experiment, this estimate was considered realistic and was further explored in the main text. The initial pathogen prevalence was increased to 40% in the tested scenarios to discern the long-term effects of pathogen prevalence. A non-zero recovery rate for *P. parva* allowed a better fit to the experimental data, suggesting that reservoir host recovery and/or immunity plays a role in the disease dynamics of *S. destruens*.

In the control experiments where there were no *P. parva* or infection, 6% of *L. delineatus* died from causes unrelated to the pathogen. This corresponded to the discrepancy between the observed and model mortality in Figure S1, where demographic rates were not included. In the main text, this was addressed by incorporating natural birth and mortality rates for all species, based on observed data.

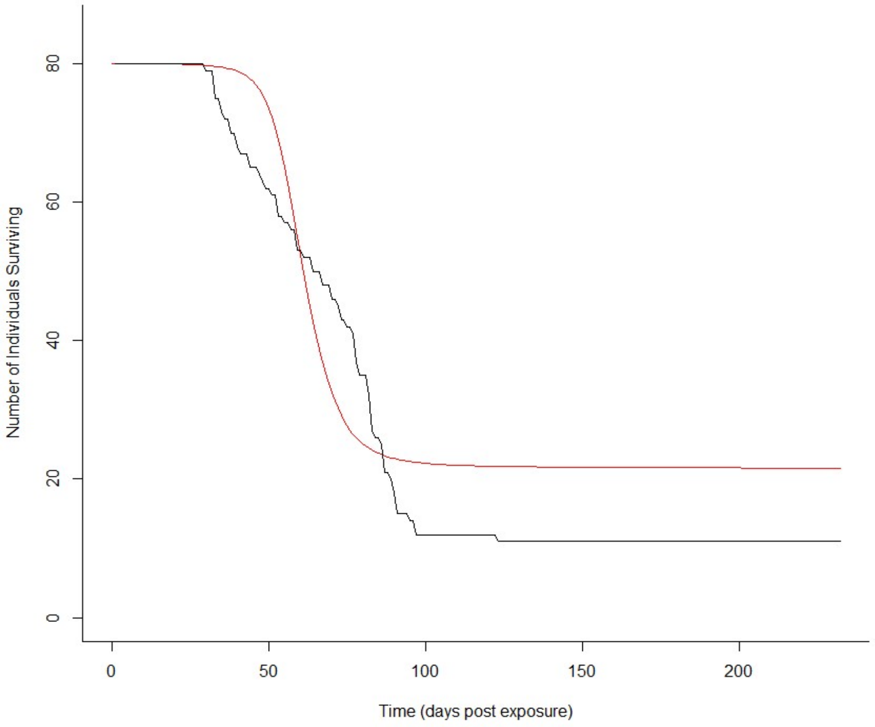

Supplementary Figure S1 Surviving population of *Leucaspius delineatus* when cohabited with introduced reservoir host *Pseudorasbora parva.* The model (red) was calibrated using experimental cohabitation data[1] shown in black.

References

1 Gozlan RE, St-Hilaire S, Feist SW, Martin P, Kent ML. Biodiversity: disease threat to European fish. *Nature* 2005; **435**: 1046.

2 Andreou D, Arkush KD, Guégan J-F, Gozlan RE. Introduced pathogens and native freshwater biodiversity: a case study of Sphaerothecum destruens. *PLoS One* 2012; **7**: e36998.

3 Al-Shorbaji FN, Gozlan RE, Roche B, Robert Britton J, Andreou D. The alternate role of direct and environmental transmission in fungal infectious disease in wildlife: threats for biodiversity conservation. *Sci Rep* 2015; **5**: 10368.

4 Spikmans F, van Tongeren T, van Alen T, van der Velde G, Op den Camp H. High prevalence of the parasite Sphaerothecum destruens in the invasive topmouth gudgeon Pseudorasbora parva in the Netherlands, a potential threat to native freshwater fish. *Aquat Invasions* 2013; **8**: 355–360.
