# Supplementary material for: The consequences of reservoir host eradication on disease epidemiology in animal communities
Source: Emerg Microbes Infect. 2016 May 11;5(5):e46–. doi: 10.1038/emi.2016.46 (PMC4893545; doi:10.1038/emi.2016.46)
Supplement: Supplementary Information 2 [file emi201646x2.pdf]

Carrying capacity

Initially, carrying capacity (K) for each species was calculated according to De Leo and Dobson's[5]allometric calculation of body weight w per km^2:

$$K = 16.2w^{-0.70} \qquad \text{Eq. S1}$$

However, given the average body sizes found for each species[6]this resulted in very small populations of *C. carpio* and *R. rutilus*to include in the model. Thus, the overall assumption that smaller species have higher carrying capacities was maintained, but the specific values for each species were estimated based on knowledge and experience of UK fish surveys and population sizes (Britton, personal communication). The values used in the model are significantly higher than those found in wild populations, but reflect heavily stocked angling habitats. Furthermore, the population size of *P. parva* was estimated at a higher level to reflect their highly invasive nature and life history[7]. These magnified values were used to uncover the role of population density in disease transmission more clearly, as smaller populations showed no significant differences between outputs. These results were also highly relevant for heavily stocked aquaculture facilities.

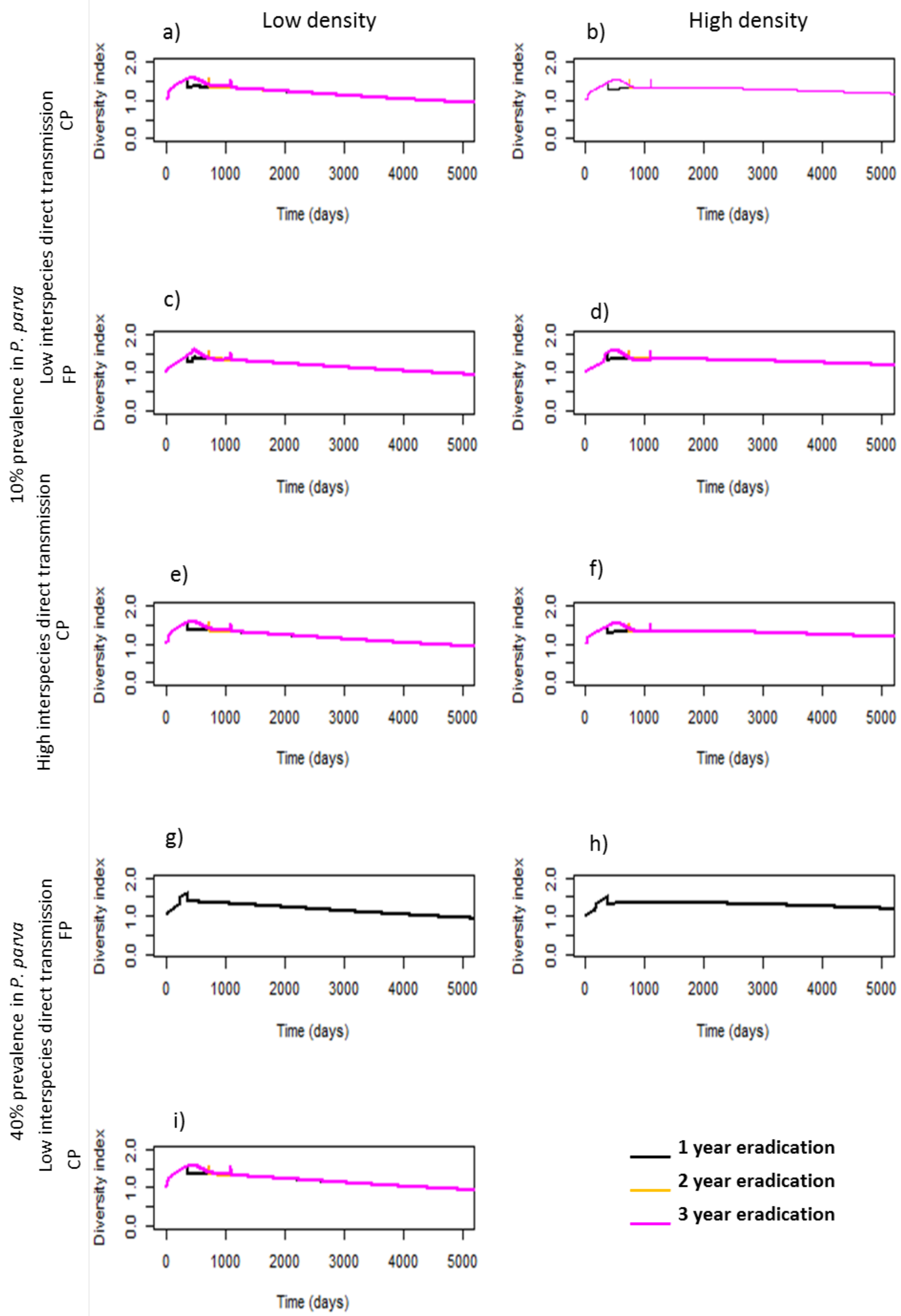
Low density
High density
a)
b)
c)
d)
e)
f)
g)
h)
i)
10% prevalence in P. parva
Low interspecies direct transmission
CP
FP
High interspecies direct transmission
CP
40% prevalence in P. parva
Low interspecies direct transmission
FP
CP
Diversity index
Time (days)
1 year eradication
2 year eradication
3 year eradication

16

Supplementary Figure S2 The Shannon biodiversity index of all scenarios over time. High density populations maintain higher levels of biodiversity than low density communities. (0.96 vs. 0.68, respectively) The proximity between introduced host and local communities affected the level of biodiversity initially (CP = close proximity; FP = far proximity). However, in the long term, communities at each density declined to similar levels of biodiversity across all scenarios.

References

5 DeLeo, Dobson AP, De Leo GA. Allometry and simple epidemic models for microparasites. *Nature* 1996; **379**: 720–722.

6 Froese, Pauly D. FishBase. www.fishbase.org. 2015.http://www.fishbase.org/ (accessed 29 Apr2015).

7 Gozlan RE, Andreou D, Asaeda T, Beyer K, Bouhadad R, Burnard2 D *et al.* Pan-continental invasion of Pseudorasbora parva: towards a better understanding of freshwater fish invasions. *Fish Fish* 2010; : 315–340.
